# Supplementary material for: Additive Manufacturing of Electrically Conductive Multi-Layered Nanocopper in an Air Environment
Source: Nanomaterials (Basel). 2024 Apr 25;14(9):753. doi: 10.3390/nano14090753 (PMC11085505; doi:10.3390/nano14090753)
Supplement: Supplementary file 1 [file nanomaterials-14-00753-s001.zip › nanomaterials-2940437-supplementary.pdf]

Supplementary Information

Additive Manufacturing of Electrically Conductive Multi-layered Nanocopper in an Air Environment

David Pervan [1,†], Anil Bastola [1,†], Robyn Worsley [1], Ricky Wildman [1], Richard Hague [1], Edward Lester [2], Christopher Tuck [1]

[1]Centre for Additive Manufacturing, Faculty of Engineering, University of Nottingham, Nottingham NG7 2RD, United Kingdom

[2]Advanced Materials Research Group, Faculty of Engineering, University of Nottingham, Nottingham NG7 2RD, United Kingdom

*Corresponding Author: Christopher.Tuck@nottingham.ac.uk

† These authors contributed equally to this work.

S1. Mean Crystallite Size Before and After Sintering

Mean crystallite size was quantified from XRD data, before and after sintering, using the Scherrer Equation:

$$t = \frac{0.9\lambda}{B_{size} \cos \theta_B}$$

Equation S1

where t is the mean crystallite size, 0.9 is the shape factor, λ is the wavelength, B_{size} is the crystallite size broadening, and θ_B is the Bragg angle.

The peak shapes were assumed to be Gaussian. It follows that:

$$B_{exp}^2 = B_{size}^2 + B_{strain}^2 + B_{inst}^2$$

Equation S2

where B_{exp} is the experimentally measured Full Width Half Maximum (FWHM), B_{strain} is the FWHM due to micro strain, and B_{inst} is the FWHM due to the instrument. All examined samples were nanoparticles; therefore B_{strain} was assumed to be negligible. In order to subtract the instrument broadening from the experimentally measured FWHM, the instrument broadening was measured using a bulk Cu reference sample. For the reference sample it can be assumed that no peak broadening due to crystallite size (crystallite sizes are sufficiently large) or micro-strains occurred and therefore only the instrument broadening is measured. The instrument broadening for the angles other than the angles of the Cu reference sample peaks were modelled. The $K\alpha_2$ contribution to scans was subtracted prior to all peak width measurements.

S2. Nano-hardness and Creep Behaviour

Indentation hardness (H_{IT}) was defined according to Meyer's Law as

$$H_{IT} = \frac{P}{A}$$

Equation S3

where P is load and A is projected area of contact. With a Berkovich indenter the projected area of the contact is given by

$$A = 3\sqrt{3}\, h_c^2 \, tan^2\theta$$

Equation S4

where h_c is the contact depth of penetration. With $\theta = 65.27°$ this equates to

Equation S5

$$A = 24.494\, h_c^2$$

Substituting this projected area function into Meyer's Law results in

$$H_{IT} = \frac{P}{24.494\, h_c^2}$$

Equation S6

It should be noted that from the unloading curve substantial elastic recovery was observed. The indentation hardness measured is not a true measure of the resistance of the material to plastic deformation but rather measures the resistance of the material to combined elastic and plastic deformations.

The creep value was obtained from a creep test. At maximum load (10 mN), the load was kept constant and displacement was measured for a time duration of 5 seconds. The creep value was calculated from:

$$C_{IT} = \frac{h_2 - h_1}{h_1}$$

Equation S7

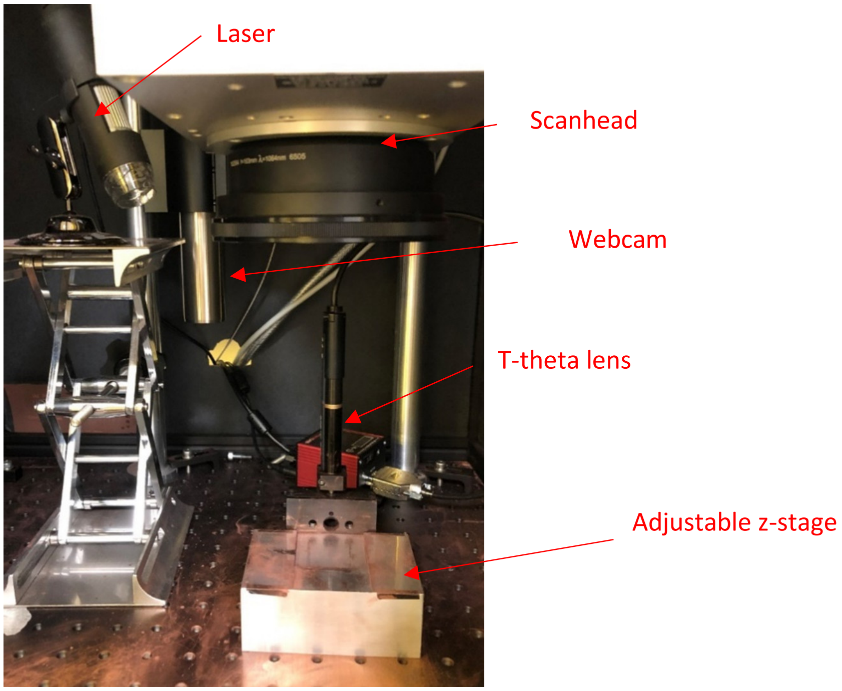

Figure S1. Bespoke **s**intering setup showing stage, camera for focussing and laser arrangement.

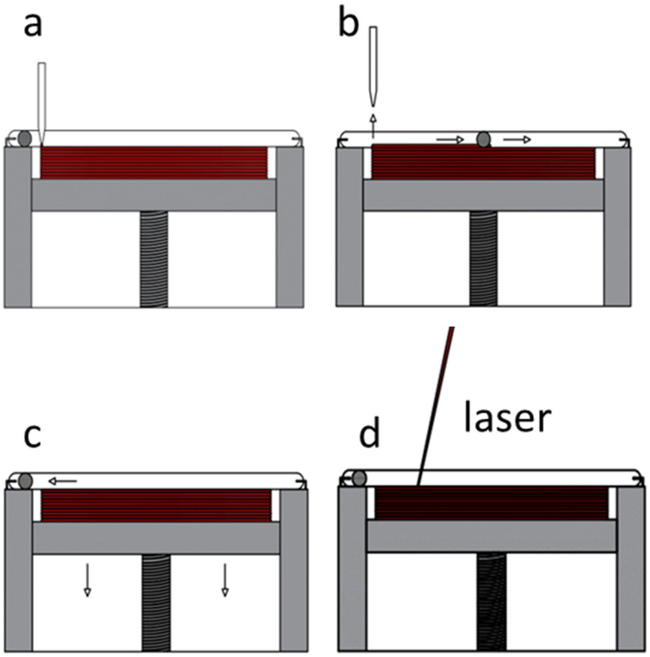

Figure S2. Diagram of the Cu NP ink deposition via bar coating and laser sintering process. a) The Cu NP ink is deposited through the nozzle onto the build platform in the case of the first layer, otherwise onto the previous layer. b) A metal bar with a wire wrapped around it moves across the build platform to spread the deposited ink to create an even layer. c) The build platform moves down and the metal bar moves back to its original position. Between c) and d) there is a time interval of 30 seconds to allow the ink to dry. d) The laser beam is focussed onto the surface through an f-theta lens which moves across the surface to selectively sinter the Cu NP layer into the previous layer.

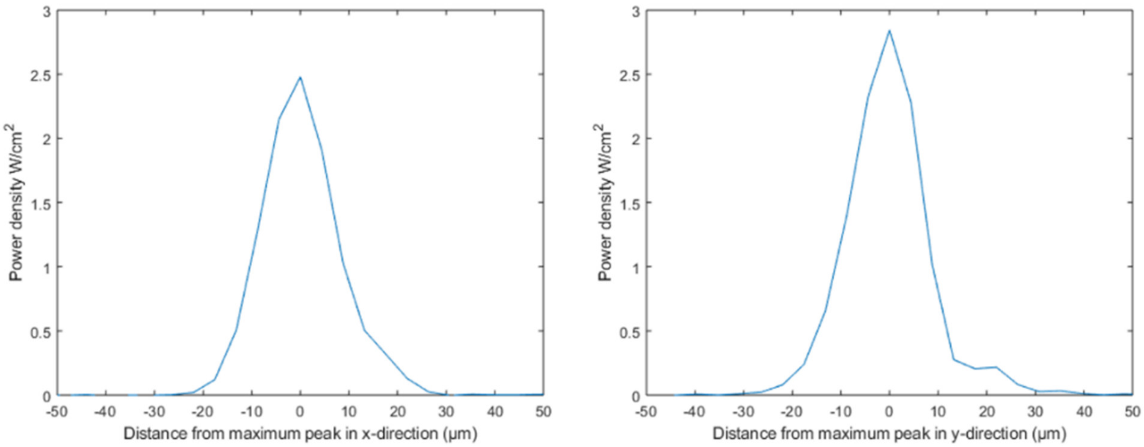

Figure S3. Laser beam power density in X-direction (left) and in Y-direction (right).

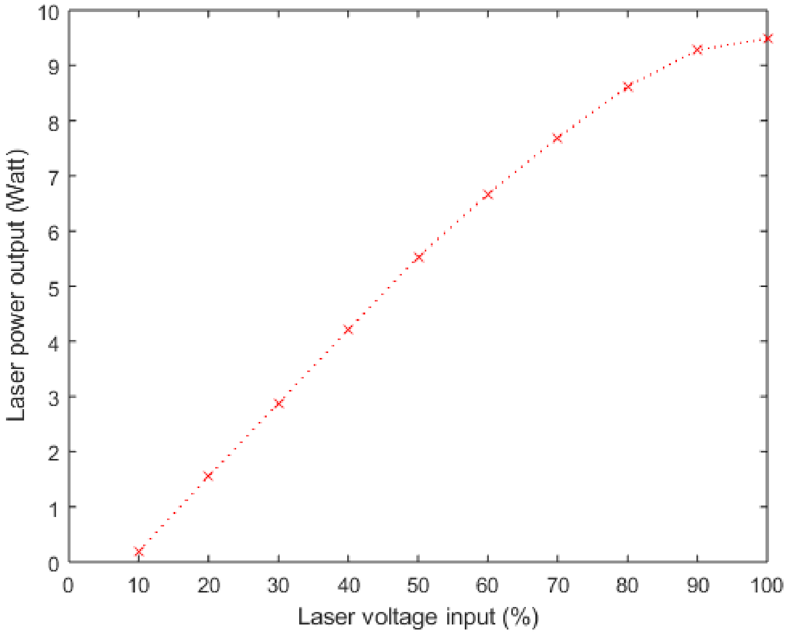

Figure S4. Laser power output against laser voltage input.

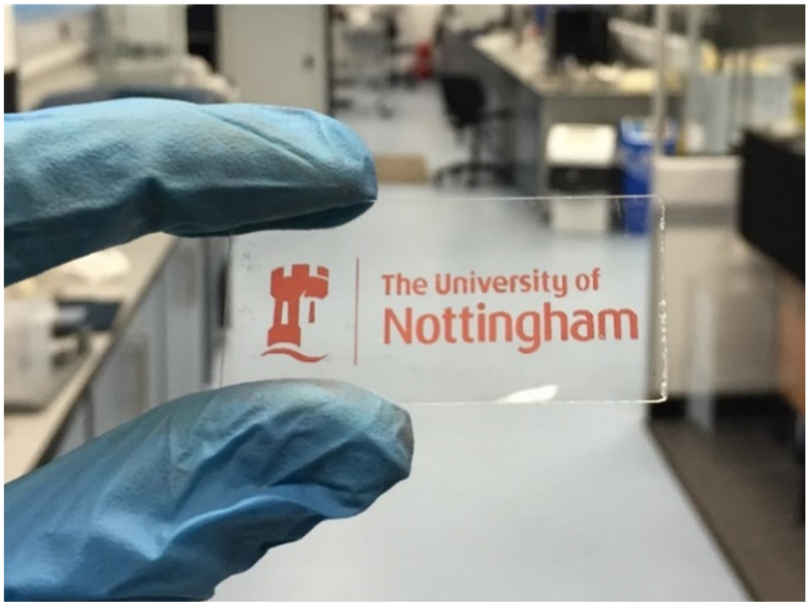

Figure S5. Inkjet-printed and sintered University of Nottingham logo using Cu NP ink.
